# Laryngeal schwannoma excised under a microlaryngoscope without tracheotomy: A case report

**DOI:** 10.3892/etm.2014.1528

**Published:** 2014-02-10

**Authors:** BAOXIN WANG, PIN DONG, BIN SHEN, HONGMING XU, JIN ZHENG

**Affiliations:** 1 Department of Otolaryngology, Head and Neck Surgery, Shanghai Jiao Tong University Affiliated Shanghai First People’s Hospital, Shanghai, P.R. China; 2 Department of Pathology, Shanghai Jiao Tong University Affiliated Shanghai First People’s Hospital, Shanghai, P.R. China

**Keywords:** laryngeal schwannoma, magnetic resonance imaging, surgical treatment

## Abstract

Schwannomas are benign encapsulated tumors arising from Schwann cells in the peripheral nervous system. Between 25 and 45% of schwannomas occur in the head and neck region. Schwannomas of the larynx are extremely rare with an incidence of 0.1–1.5% in all benign laryngeal tumors. Laryngeal schwannomas usually occur in females aged between their 4th and 5th decades. The most successful curative method is surgical resection. When selecting the surgical method, the size and location of the tumor, as well as the requests of the patient, should be considered. The present case report describes a 29-year-old female patient with symptoms of hoarseness and dyspnea on exertion. Through endoscopic biopsy, histopathology revealed a schwannoma. Considering the symptoms, age and preferences of the patient, the method of trans-oral microlaryngoscopic excision without tracheotomy was used to excise the tumor located in the aryepiglottic fold. Results from a short-term follow-up showed the postoperative result to be satisfactory.

## Introduction

Schwannomas are solitary, encapsulated, slow-growing, benign tumors arising from Schwann cells of the peripheral nervous system (1). Between 25 and 45% of all schwannomas occur in the head and neck region and are usually located in the parapharyngeal space. Schwannomas of the larynx are extremely rare, accounting for 0.1–1.5% of all benign laryngeal tumors (2). The current report presents a case of laryngeal schwannoma located in the aryepiglottic fold.

## Case report

A 29-year-old female consulted the Department of Otolaryngology, Head and Neck Surgery, Shanghai Jiao Tong University Affiliated Shanghai First People’s Hospital (Shanghai, China) with symptoms of hoarseness and dyspnea on exertion that had been present for 3 years. The patient had also experienced dysphagia for 1 month, which had recently worsened. In a previous hospital, 2 months prior to consultation, the tumor had been biopsied and the pathodiagnosis revealed a schwannoma. No other significant past medical illnesses were present and systemic examination was normal. No palpable lymph nodes or café au lait patches were present. Informed consent was obtained from the patient.

**Figure 1:**
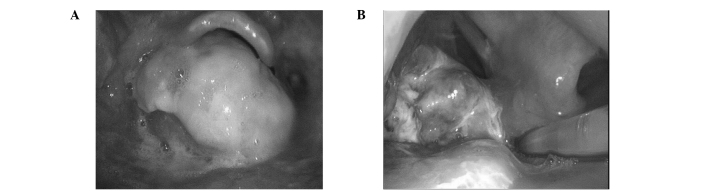
Indirect laryngoscopic view following (A) biopsy and (B) surgery.

**Figure 2:**
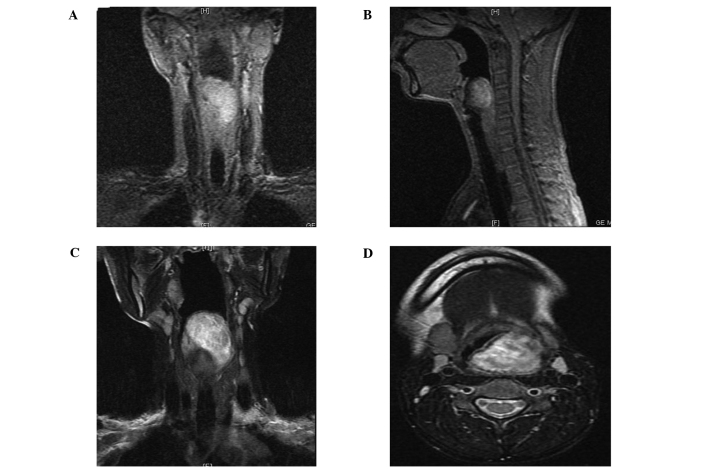
MRI scans from a 29-year-old female. T1-weighted (A) coronal section of the larynx and (B) sagittal section of the larynx. T2-weighted (C) coronal section of the larynx and (D) axial section of the larynx. MRI, magnetic resonance imaging.

Outpatient laryngoscopy revealed a large mass located within the left recessus piriformis. In addition, the glottis was obstructed and the vocal folds were not visible (Fig. 1A). Magnetic resonance imaging (MRI) of the lesion revealed it to be isodense compared with muscle in the T1-weighted images that exhibited strong, inhomogeneous enhancement by gadolinium. In the T2-weighted images, the lesion was shown to be hyperintense and inhomogeneous. The lesion was a well-defined, enhanced, inverted conical mass, measuring 58×29×26 mm in size and centered on the posterior and lateral wall of the left recessus piriformis. The lesion appeared to originate from the left aryepiglottic fold. The left recessus piriformis and vocal cords were effaced (Fig. 2).

**Figure 3:**
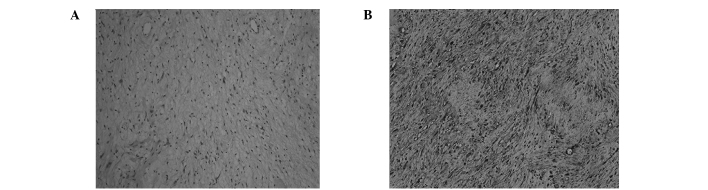
(A) Histopathological specimen shows the characteristic Antoni A pattern of the tumor with pallisade-like structures and a loose cellular Antoni B pattern. (B) Immunochemical staining evaluation demonstrates the presence of S-100 protein posivity (H&E staining; magnification, ×100).

Following consideration of age and symptoms, the patient selected not to undergo surgery via an incision in the lateral neck. Therefore, a trans-oral resection using a microlaryngoscope was performed and postoperative tracheostomy was avoided. Treatment for the symptoms and nutritional support was administered, including anti-inflammatory agents, hemostatic methods and nasogastric nutrition. Microscopic examination showed compact cellular margins and prominent palisade formation, known as Antoni A patterns. A less cellular, loosely-textured pattern, known as the Antoni B pattern, was also observed. Immunochemical staining evaluation demonstrated the expression of S-100, resulting in a final diagnosis of a benign schwannoma (Fig. 3).

The patient was discharged on the fifth postoperative day and the nasal feeding tube was removed. Postoperative laryngoscopy showed that the left vocal cord was immobile and that the remaining tumor was present within the left recessus piriformis (Fig. 1B). It is unlikely that the immobility of the left vocal fold was the result of injury. The nerve may have caused the immobility. Close follow-up of the patient was advised.

## Discussion

Schwannomas and neurofibromas are two types of neurogenic tumor. Neurofibroma is the main differential diagnosis of laryngeal schwannoma. Other differential diagnoses include chondroma, adenoma, laryngeal cyst and laryngocele (3). Schwannomas account for 0.1–1.5% of all benign laryngeal tumors. Schwannomas are encapsulated, benign, solitary tumors that grow slowly and were first reported by Verocay in 1908 (4,5). Schwannomas deriving from perineural Schwann cells grow extrinsically to their parent nerve fascicles and may develop along any somatic or sympathetic nerve in the body (with the exception of the olfactory and optic nerves due to the lack of Schwann cell sheaths) (6). By contrast, neurofibromas originate from perineural fibrocytes, involving nerve fibers and sheath cells. These tumors exhibit diffuse proliferation and are usually intertwined with the nerve trunk (2).

Schwannomas most commonly occur in females between the ages of their 4th and 5th decades. In total, 80% of laryngeal schwannomas are found in the aryepiglottic folds, while the remaining 20% are found in the false and true cords (7). In the majority of cases, the nerve of origin is likely to be the internal branch of the superior laryngeal nerve (8).

Clinical symptoms are related to the size and location of the tumor (1). The patient may complain of a number of symptoms, including dysphagia, dyspnea, dysphonia, hoarseness and a foreign body sensation in the throat (3). However, these clinical features are meaningless to the definite diagnosis (1).

Laryngoscopic evaluation reveals a round submucosal mass originating from the aryepiglottic fold and/or true and/or false vocal cords.

With CT scans, a small schwannoma is regarded as a homogenous, enhanced mass (9). When the size is large (>3 cm), tumors are often heterogeneous, with randomly distributed areas of low attenuation, surrounded by a peripheral ring of enhancement. Generally, cystic components may be observed (10).

When examined by MRI, T1-weighted images of schwannomas have a low signal intensity ranging between those of the brain and muscles, with a homogeneous or heterogeneous appearance (11). With T2-weighted images, the schwannomas have a brighter signal than cerebrospinal fluid and may be heterogeneous or homogeneous (11). The images are usually well-enhanced following gadolinium injection (9).

Although CT and MRI scans reveal a well-defined submucosal mass without surrounding tissue destruction (12), the methods are not able to differentiate a schwannoma from other laryngeal neoplasms (13). Histopathological examination is the gold standard. The diagnosis of schwannoma may be made with the presence of three features: i) a clear capsule; ii) the presence of Antoni A and/or B areas; and iii) a positive reaction for S-100 protein (14). Antoni A regions are described by densely packed spindle cells with nuclei aligned in parallel rows in a palisade pattern. Antoni B regions consist of loosely arranged spindle cells, with vacuoles and spindle-shaped nuclei prone to degeneration (2).

Laryngeal schwannoma is a rare benign tumor and the curative method is surgical resection (13). However, this method may not be suitable for every patient due to anatomical constraints and the requests of patients (15). There are a number of surgical methods that may be used, including trans-oral microlaryngoscopic excision, median thyrotomy and lateral pharyngotomy. Generally, the treatment of laryngeal schwannomas depends on the location and size of the tumor (16). Trans-oral microlaryngoscopic excision is suitable for small lesions and successful resections have been reported (3). In 2011, Kayhan *et al* (12) demonstrated the first case of transoral robotic surgery-assisted excision of a schwannoma in the supraglottic larynx. An open approach may be wise for larger lesions (13). The complete excision of the tumor is desirable, and recurrence is very rare, even if a portion of the capsule is left behind (4). For these reasons, the method of trans-oral microlaryngoscopic excision was used for the patient in the current case. Since schwannomas are slow-growing, we are able to excise the tumor under a microlaryngoscope without tracheotomy to maintain the patient’s quality of life.
